# Colleges and Associations as Guards of Our Standing among the Professions1Presented at the A. V. M. A. Meeting, Atlantic City, September, 1901.

**Published:** 1901-12

**Authors:** L. Van Es

**Affiliations:** Mobile, Ala.


### COLLEGES AND ASSOCIATIONS AS GUARDS OF OUR STANDING AMONG THE PROFESSIONS.[1]

By L. VAN ES, M.D., V.S.,
MOBILE, ALA.

LIVING as we do in a land in which horses, mules, cattle, sheep, and hogs are counted by the millions; in a land which is gradually developing to be the source of supply of animals for food and carrying purposes for the civilized world; in a land in which

[1] Presented at the A. V. M. A. Meeting, Atlantic City, September, 1901.

countless numbers of its citizens are engaged in and dependent on the exploitation of these enormous resources, we cannot fail to see that our profession entered into the new century under the most favorable auspices. Indeed, the field of usefulness of the modern veterinarian extends in many directions. Numbers of us are engaged in the practice of our art in civil life and the army ; others in the employ of our Federal Government stand as sentinels, so that the reputation of our meatstuffs abroad shall not suffer from the exportation of diseased parts or from the malice of foreign competitors ; many in the employ of the States and the union watch that our wealth of live-stock shall not be endangered by the introduction of scourges from without or from the propagation of diseases endemic within ; others, again, are in charge of the milk- and meat-inspection service of some of our cities ; a few, favored by opportunity and ability, are engaged in original research in the great field of animal pathology, in order that disease may be better understood and more rationally treated ; while others have the noble task of educating our youth—either our young farmers and stock-raisers at the agricultural institutions or our prospective colleagues at the veterinary colleges.

With a few exceptions, perhaps, there usually is a fair compensation attached to these various occupations in which veterinarians are active ; in fact, the profession has entered on an era of prosperity which, compared to its condition of a few years ago, must give us cause to rejoice.

Without doubt, the better times are factors of great encouragement to the individual workers in the ranks, but they also tend to have a beneficial influence on the profession as a whole.

Now that the days of empiricism are wellnigh counted, and the rewards of the practitioner are such as to place most of the comforts of life at his disposal, we may have noted that many young men of a greater ability than before constitute the bulk of the forces from which our profession is recruited. As a consequence the profession is gradually growing in prestige and esteem by our fellow-citizens—growing, I say, as we are not yet full-grown in standing among the other professions which are adults in public consideration.

As a sign that we have not yet reached universal recognition, I may mention the rebuke suffered by the veterinary profession at the hands of the higher house of our national legislature, in spite of the most vigorous efforts of our army committee—composed of men of the highest stamp and standing—and of those legislators who so nobly championed our cause. Whatever causes contributed to bring about our defeat, it can be boiled down to the fact that the majority of the Senate denied us the recognition which the profession asked for. It may have been ignorance of our usefulness; it may have been ignorance of the fact that our profession contains a very large number of able, earnest workers, which caused Senators to say and think unpleasant things about us and to bring about the defeat of the Army bill. But, let me ask, is it not possible that such a Senator had been prejudiced by what little he actually did see of the profession? Perhaps he once had occasion to observe a veterinarian engaged in the task of impressing on a horseowner's mind the fact that in order to keep his horse from having colic, worms, or scratches he should allow him to file the animal's teeth; or perhaps even his home town is plastered with posters telling him of all the great things Dr. So and So can do in the way of animal surgery, and how his star remedy cures diseases ranging from heaves to infectious abortion; and yet those men may be graduates from chartered veterinary colleges and have diplomas entitling them to degrees just like those of the most honorable members of the profession.

If it so happens that such is the only acquaintance our Senator has with the profession, how can we expect him to indorse our requests, as Senators are just as liable to become biased as ordinary mortals? I do not wish to say that such methods are at all common among graduate veterinarians, but careful observation in a good many cities will cause one to admit of the existence of such practitioners who could be classified as graduate quacks. Nor do I contend that they damage the standing of our profession only in the eyes of Senators, but their evil influence is of a general character, and by no means increases our professional respectability. It is evident that the elimination of such undesirable elements from the profession is of no small importance; and with this in view let us ask, What remedies could be applied against them?

It is, of course, impracticable to reach such men by legislative or other drastic measures; but the case calls more for prevention than for a cure, and by prevention in this instance I mean (1) a careful sifting process applied to the prospective elements of the profession, and (2) the universal adoption of the highest standard on the part of the profession itself—the former to be accomplished by our colleges, the latter by means of the associations.

In speaking of a sifting process carried on by the colleges, I mean the testing of the mental and moral capacity of their matriculates before admission and before granting them diplomas; this not merely as a matter of form, but an actual gleaning after the test has been made, by which persons found to be non-proficient be turned back until they will be able to come up to the standard. In other words, let colleges assume the responsibility not only for the technical ability but also for the ethics of their graduates.

Such a process, rigidly carried out, would insure a greater percentage of desirable members to the profession, and men admitted on this plan will not be so liable, through incompetence, to resort to business methods unbecoming a professional man. Men may be led from the path of professional virtue by excessive commercialism or greed, but I regard that incompetence and lack of skill account for more charlatans than any other cause.

Aside from being careful in the selection of their students, colleges can also exert a most elevating influence by bringing their teaching facilities up to the highest standard of efficiency; and it is encouraging to note that some of our veterinary schools have already attained a completeness in organization of faculty and equipment of laboratories and clinics undreamed of some years ago.

But all our colleges are not equally well equipped to dispense learning to our future brothers, and it is interesting to note the contrast between individual institutions.

While most of our colleges require an attendance during three sessions of from six to nine months each, others claim to turn out competent veterinarians in two sessions of five months, in spite of the fact that the amount of information and practical skill supposed to be in possession of a modern veterinarian is of such a magnitude that it is wellnigh impossible to acquire it in so short a period. Some schools insist that their matriculates shall give evidence of a higher education, others regard the cash amount of their tuition fee as the only requirement for admission, and let educational qualifications go by default, thereby flooding a supposed learned profession with numbers of unlearned men.

There are colleges whose only purpose is to educate, and others which are simply commercial enterprises, or which serve only to give a certain amount of prominence to their promoters; and in regard to the latter class, I beg to suggest that they can do no greater service to the profession than by retiring from business if it is impossible for them to acquire more honorable motives or come up to a higher standard.

Colleges whereat veterinary medicine and surgery and all that pertains thereto is taught by veterinarians and not by medical men, however learned—colleges whose faculties are thorough in their teachings, and have the proper number of sessions of a sufficient duration to do it in—can do more toward elevating our standing than all sorts of legislative measures, however useful they may be under certain circumstances. Greater efficiency of colleges will always be followed by greater efficiency of their alumni and by higher standing of the profession, however slow the evolution may be. Let us, therefore, look to those of our colleges which educate as the bulwarks of our profession against the invasion of incompetent and undesirable elements.

A factor of equal importance in the upholding of our professional dignity is found in the associations. Not only are association meetings of great value to their members as a means of education, but the fact that most associations prescribe to their members how to conduct themselves in their business methods singles out association members as men with a good conception of professional ethics; and let it here be said that no matter to what ethics have degenerated in the commercial world, it still is one of the strongest factors of professional dignity. In a great many instances associations owe their origin to a movement to antagonize empiricism, and in this struggle they have by their victories saved an enormous wealth to our country, prevented or cut short great torture and suffering to our domestic animals at the hands of quacks, and placed the profession in a better light.

They have, by concerted action of their members, caused legislation to be enacted favorable to the profession, not only by putting a check to empiricism, but also in forcing the public to recognize it as something above a mere trade of dehorners, toothfilers, or gelders; as something in which action of brain and skill of hands should place its members on an equal plane with those who minister to suffering mankind.

Notwithstanding the great good already accomplished, their usefulness in bringing about professional greatness could still be increased if the large number of eligible practitioners who are not members could be made to understand that they are withholding their support from a cause which has so much to do with their own welfare.

Perhaps the power of veterinary associations, along with greater membership, could still be increased by a more perfect organization; and here I refer to conditions in Continental Europe, where it is often seen that local societies are divisions of a national body, and are thereby in a position to develop greater strength at any one point. Whether this plan would be desirable on our continent, I cannot say, but perhaps it is worth considering. If such a perfect organization could be made practicable in this country the local State associations, as divisions of and backed by the national body, could perhaps be instrumental in preventing the issue of charters to worthless colleges in their respective States, and help to cut short the eruption of institutions which, by their inefficient teaching facilities or staffs, their short courses, or the low grade of their matriculates, do much toward making us impossible as a respectable profession.

For the sake of our professional integrity, let us hope that all respectable veterinarians will become association members; that they always will be wise in the selection of their officers, and ever further the advance of the profession by striving for greater knowledge and skill in their work, by making their business methods the most straightforward and most honorable, and by being ever watchful that no undesirable elements shall invade it.

